# Nitrate Accumulation and Leaching in Surface and Ground Water Based on Simulated Rainfall Experiments

**DOI:** 10.1371/journal.pone.0136274

**Published:** 2015-08-20

**Authors:** Hong Wang, Jian-en Gao, Xing-hua Li, Shao-long Zhang, Hong-jie Wang

**Affiliations:** 1 Institute of Soil and Water Conservation, Chinese Academy of Sciences and Ministry of Water Resources, Yangling, Shaanxi Province, China; 2 Institute of Soil and Water Conservation, Northwest A&F University, Yangling, Shaanxi Province, China; 3 University of Chinese Academy of Sciences, Beijing, China; 4 College of Water Resources and Architectural Engineering, Northwest A&F University, Yangling, Shaanxi Province, China; Glasgow University, UNITED KINGDOM

## Abstract

To evaluate the process of nitrate accumulation and leaching in surface and ground water, we conducted simulated rainfall experiments. The experiments were performed in areas of 5.3 m^2^ with bare slopes of 3° that were treated with two nitrogen fertilizer inputs, high (22.5 g/m^2^ NH_4_NO_3_) and control (no fertilizer), and subjected to 2 hours of rainfall, with. From the 1st to the 7th experiments, the same content of fertilizer mixed with soil was uniformly applied to the soil surface at 10 minutes before rainfall, and no fertilizer was applied for the 8th through 12th experiments. Initially, the time-series nitrate concentration in the surface flow quickly increased, and then it rapidly decreased and gradually stabilized at a low level during the fertilizer experiments. The nitrogen loss in the surface flow primarily occurred during the first 18.6 minutes of rainfall. For the continuous fertilizer experiments, the mean nitrate concentrations in the groundwater flow remained at less than 10 mg/L before the 5th experiment, and after the 7th experiment, these nitrate concentrations were greater than 10 mg/L throughout the process. The time-series process of the changing concentration in the groundwater flow exhibited the same parabolic trend for each fertilizer experiment. However, the time at which the nitrate concentration began to change lagged behind the start time of groundwater flow by approximately 0.94 hours on average. The experiments were also performed with no fertilizer. In these experiments, the mean nitrate concentration of groundwater initially increased continuously, and then, the process exhibited the same parabolic trend as the results of the fertilization experiments. The nitrate concentration decreased in the subsequent experiments. Eight days after the 12 rainfall experiments, 50.53% of the total nitrate applied remained in the experimental soil. Nitrate residues mainly existed at the surface and in the bottom soil layers, which represents a potentially more dangerous pollution scenario for surface and ground water. The surface and subsurface flow would enter into and contaminate water bodies, thus threatening the water environment.

## Introduction

Nitrate is a common contaminant of surface water and groundwater and it can cause health problems in infants and animals as well as eutrophication of water bodies [1–7]. The World Health Organization and the U.S. Environmental Protection Agency have established a maximum contaminant level for nitrate of 10 mg/L as NO_3_^-^–N in drinking water [8–10]. Many studies have shown that agricultural activities are a significant source of surface and ground water pollution due to long-term and excessive fertilizer use [7, 11–16].

Non-point source pollution caused by nitrogen from agro-ecosystems is a serious threat to water environments and has received increasing attention regionally and globally [12, 16–20]. Agricultural activities contributed to approximately 75% of non-point pollution, which accounted for approximately two-thirds of the total pollution, in the US [21]. Agriculture is a primary source of river and groundwater pollution in rural areas of the UK [22, 23]. The total nitrogen provided by agricultural non-point sources reached approximately 60% of the total water pollution in the Netherlands [24]. Approximately 94% of the nitrogen loading in 270 rivers was caused by non-point source pollution in Denmark [25]. Since the 1980s, nitrogen fertilizer consumption in China has substantially increased, and nitrate pollution of drinking water has become a serious problem [26]. Fan and Hao [27] summarized the primary factors for the accumulation and leaching of NO_3_^-^–N in a soil profile and its potential contamination in surface and underground water in northern China.

A number of studies have shown that nitrate-nitrogen (NO_3_^-^–N) loss through subsurface drainage is a major source of pollution for surface and groundwater bodies, thus threatening the water environment [28–31]. Nitrate is both soluble and mobile, it is prone to leaching through soil with infiltrating water, and it can persist in shallow groundwater for years [32]. Moreover, the hydrogeological settings, seasonal trends and anthropogenic activities are major factors that influence the mobility and accumulation of nitrates [33]. Under rainfall or irrigation conditions, high levels of soluble nitrates (NO_3_^-^–N) leak through soil and into groundwater and then drain away with the groundwater flow. In the Weihe River Basin, groundwater is a streamflow recharge source in the upper reaches; in the middle reaches, one side of the river flow supplies the groundwater, and on the other side, the groundwater supplies the flow [34]. Therefore, nitrate leakage can cause nitrate pollution of groundwater; subsequently, the contaminated groundwater is likely to drain into rivers, resulting in further environmental damage to surface water [35].

Monitoring and modeling approaches have been used to study nitrate contamination in surface water and groundwater. Feng et al. [36] studied the effects of different levels of rainfall and fertilization on the soil nitrate distribution and the cumulative amount of nitrate in maize through simulated rainfall field experiments in Shunyi of Beijing, China. Chen et al. [37] studied the nitrate vertical transport rule in farmland soil through soil column and field experiments. Huang et al. [38] investigated the transforming behaviors and removal efficiencies of NO_3_^-^–N in river bank filtration using two soil-body filtration experiments. The SWAP and ANIMO models were used to simulate the transport of water, nitrate and phosphorus nutrients, during intense rainfall events generated by a simulator, and during natural rainfall [39]. The HYDRUS-1D model was used to simulate the movement of Br. as a tracer of surface-applied N fertilizer, and nitrate remaining in the soil profile under conditions of heavy rainfall and high-intensity irrigation [40]. Based on the results and analysis of the soil water atmosphere plant model (SWAP) or DRAINMOD (DM) models, Wang et al. [35] developed a mechanistic model of nitrogen transport and transformation in farmland soil that was suitable for organic and inorganic fertilizer application. The soil and water assessment tool (SWAT) has also been used to simulate the land phase of the hydrological cycle, as well as to obtain streamflows, groundwater recharge, and nitrate (NO_3_^-^) load distributions in various components of runoff [41]. Wriedt and Spindler [42] simulated the steady state, transient flow andnitrate transport using MODFLOW and MT3DMS, driven by average and monthly lysimeter data of recharge and nitrate leaching. The hydrological SWAT model was integrated with the modular finite difference groundwater flow model (MODFLOW) and the modular 3-dimensional multi-species transport model (MT3DMS) to obtain groundwater flow and NO_3_^-^–N transport [41]. The monitoring approach can more directly estimate the effects of nitrate contamination on surface and soil, but it is difficult to study the effect on groundwater pollution. Therefore, the the use of modeling approaches to study groundwater pollution or the interaction between surface water and groundwater has become a trend. However, the accuracy of the modeling results greatly depends on the accuracy of the information and on the magnitude and distribution of aquifer permeability [43].

Based on an independently developed experimental system that integrates surface water, soil infiltration and groundwater experimental equipment, this study conducted simulated rainfall experiments to evaluate nitrate accumulation and the leaching process in surface water and groundwater.

## Materials and Methods

### Experimental equipment and conditions

**Fig 1:**
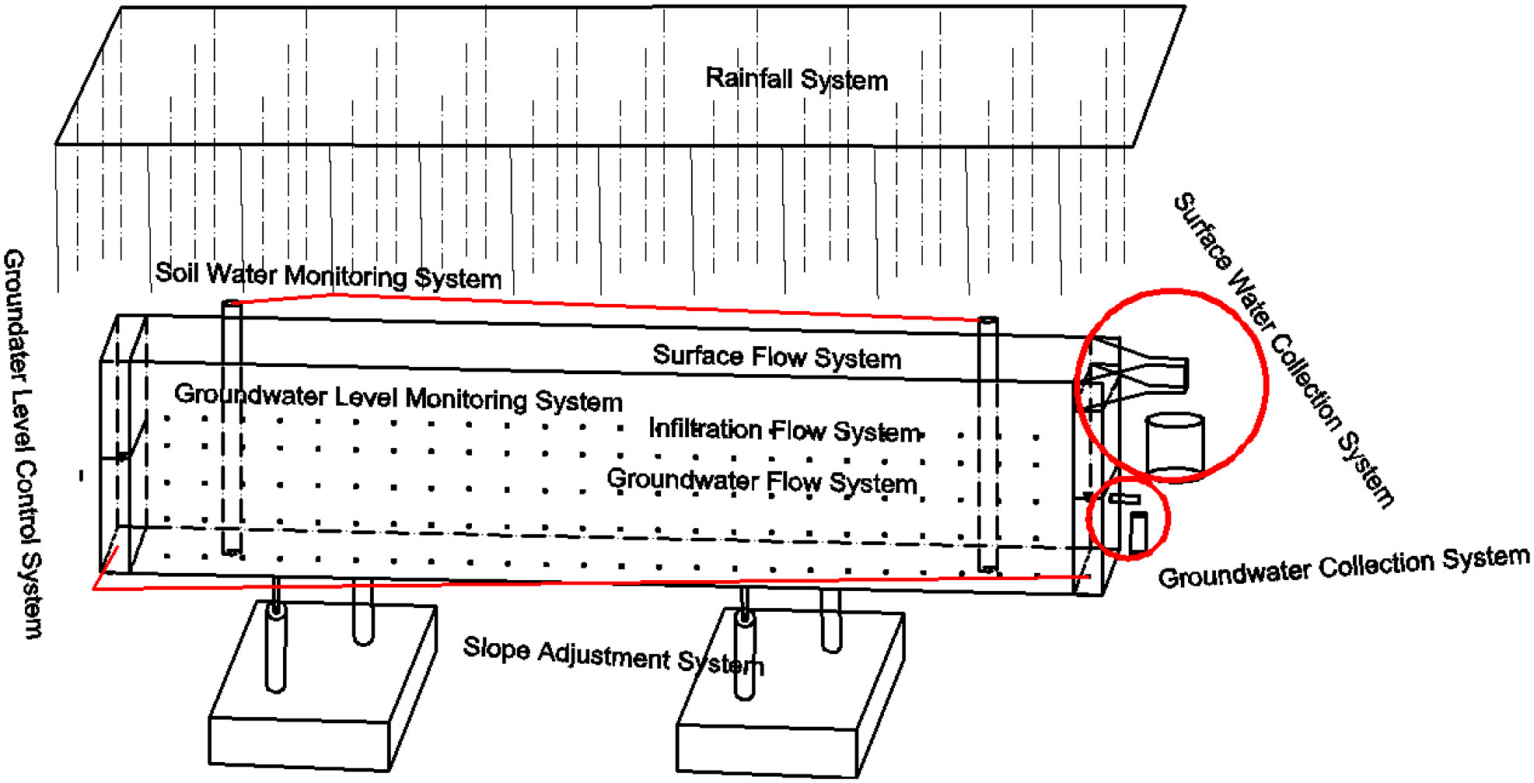
Experimental equipment.

The simulated rainfall experiments were performed in the Rainfall Simulation Hall of the State Key Laboratory of Soil Erosion and Dryland Farming on the Loess Plateau of China in 2013. The experimental equipment consisted of 10 systems (Fig 1). From the top to the bottom, the equipment configuration consisted of the rainfall system, surface flow system, infiltration flow system, groundwater flow system and slope adjustment system. From left to right, the configuration was the groundwater level control system, soil water monitoring system, groundwater level monitoring system, surface water collection system and groundwater collection system. The soil box was 5.3 m long, 1 m wide and 1 m deep. Soil water was monitored using a neutron probe (Diviner 2000) [44], and the slope was 3° in this study.

The rainfall system had an automatic simulation device consisting of an under-sprinkler providing uniform rainfall conditions. The nozzles in the system were located approximately 16.5 meters above the underlying surface. They could be set to any preselected rainfall intensity from 15 to 180 mm/h. The average rainfall intensities of each experiment are shown in Table 1, and the mean rainfall intensity of the twelve experiments was 65.7 mm/h. The rainfall duration was 2 hours for each experiment, and the cumulative rainfall was 1576.4 mm.

**Table 1:** Average rainfall intensity of each experiment (mm/h).

Number	1	2	3	4	5	6	7	8	9	10	11	12
Rainfall Intensity	75.4	84.6	80.3	60.8	55.6	56.5	56.0	63.8	65.0	66.0	63.1	61.1

**Fig 2:**
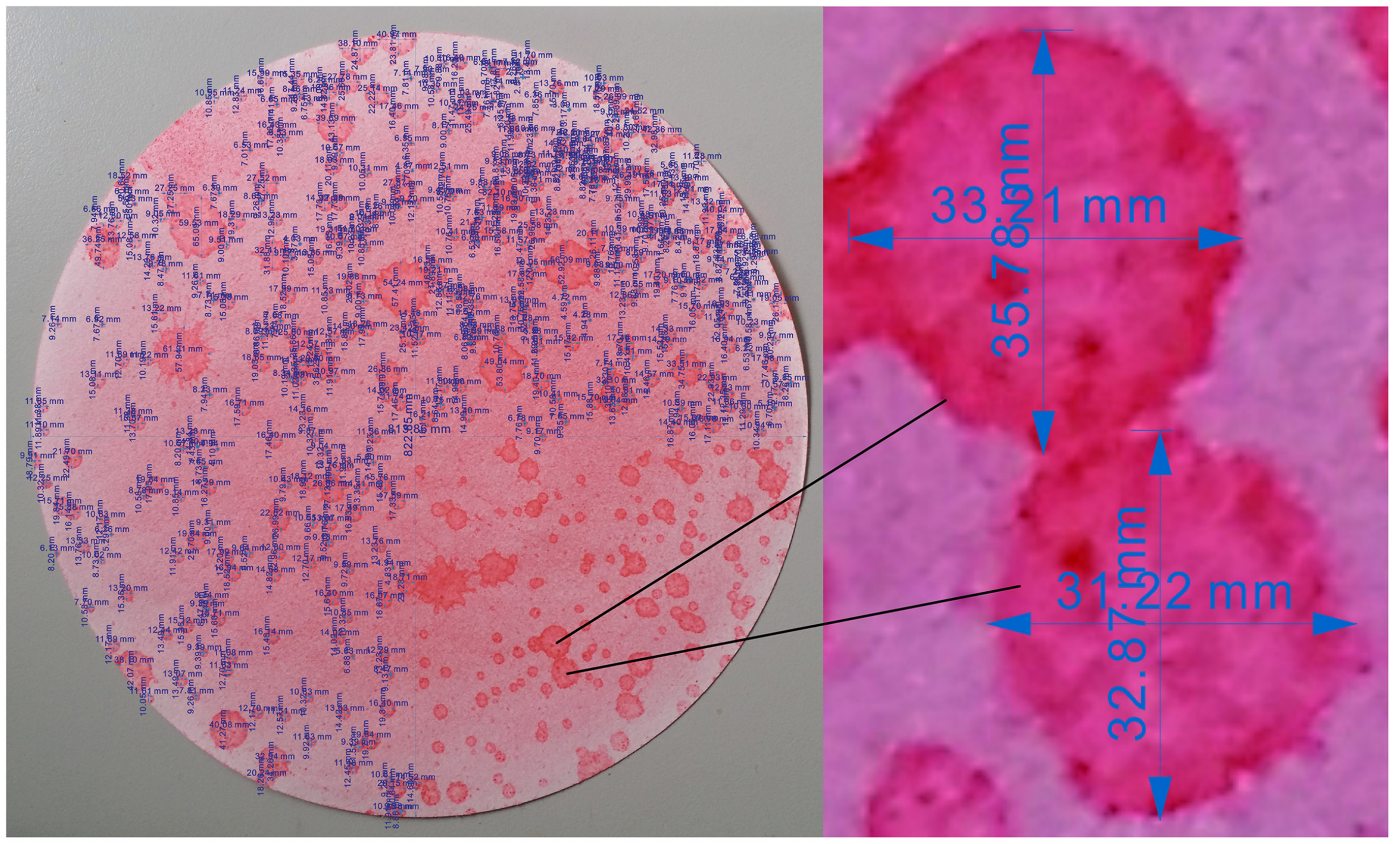
Measurement of the stain diameters based on the crossing method (75 mm/h).

**Fig 3:**
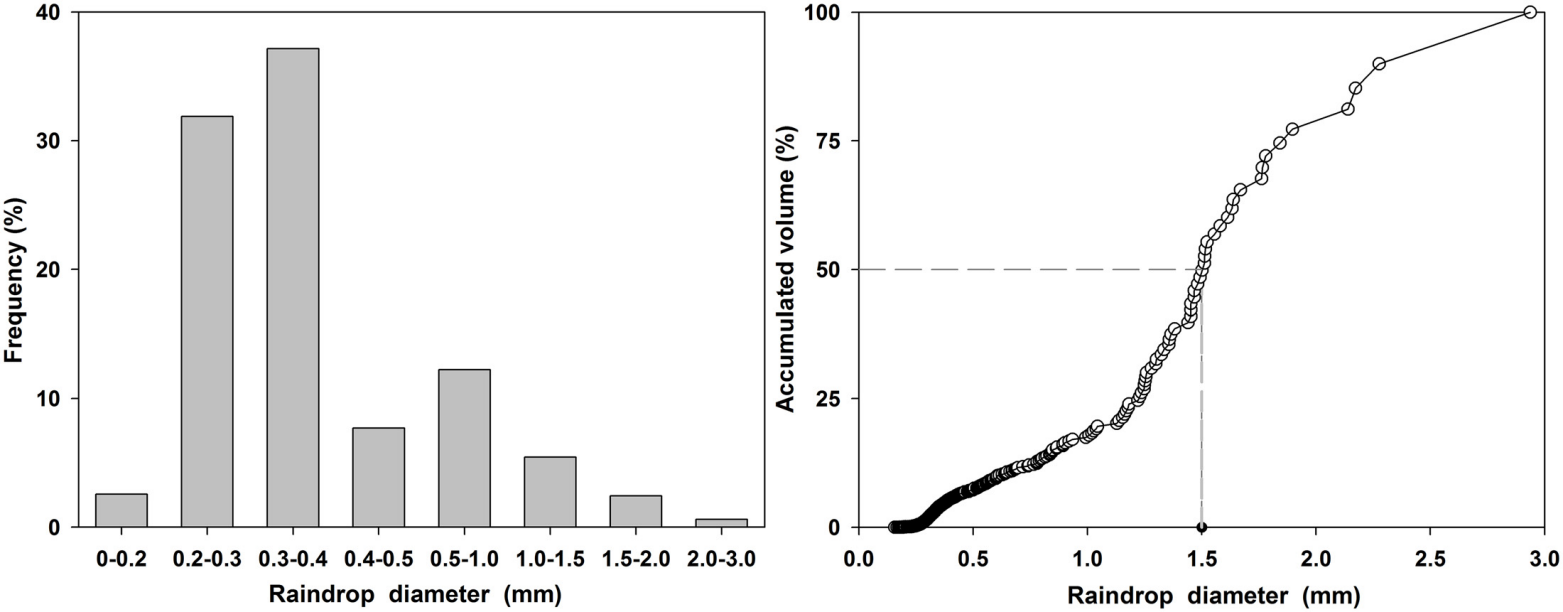
Raindrop diameter vs. frequency and the relationship between the accumulated volume and raindrop diameters.

Raindrop size distribution and kinetic energy are two important factors for rainfall infiltration and groundwater recharge [45–49]. The stain method [45, 50, 51] was used in this study to measure the sizes and distributions of the raindrops [52]. CorelDRAW software was used to measure the horizontal and longitudinal diameters of the stains with the crossing method, and the results from the experiment with a rainfall intensity 75 mm/h are presented in Fig 2. Then, the stain diameters were measured as the average value of the horizontal and longitudinal diameters. Based on the relationship (Eq 1) between the drop size and stain size [51], the raindrop diameters were calculated as follows:

$$d=0.36D^{0.73}$$ (1)

where d is the raindrop diameter (mm), and D is the stain diameter (mm). A histogram of the raindrop diameter (d) vs the frequency and the mid-values of the raindrop diameters (d_50_) obtained from the relationship between the accumulated volume and raindrop diameter were analyzed to determine the raindrop size distribution (Fig 3). Subsequently, the fall velocities of the raindrops were calculated using different formulas according to the raindrop sizes. When the raindrop diameter was less than 1.9 mm, the improved Sha Yuqing formula [53, 54] was used to calculate the velocity (Eq 2):

$$v=0.496\times 10^{\left[\sqrt{28.32+6.524lg0.1\;d\text{-}{\left(lg0.1d\right)}^{2}}-3.665\right]}$$ (2)

where v is the fall velocity of the raindrop (m/s).

When the raindrop diameter was equal to or greater than 1.9 mm, the fall velocity of the raindrops was calculated using the improved Newton formula [55] (Eq 3):

$$v=(17.20-0.844d)\sqrt{0.1d}$$ (3)

The total kinetic energy of the raindrops on the filter paper can be calculated using the following formula (Eq 4):

$$e=\overset{1}{\underset{i}{\sum }}e_{i}=\overset{1}{\underset{i}{\sum }}\frac{1}{2}m_{i}v_{i}^{2}=\frac{1}{12}\overset{1}{\underset{i}{\sum }}\pi d_{i}^{3}\rho v_{i}^{2}$$ (4)

where e is the total kinetic energy of the filter paper (J), i is the number of the raindrop, *e*_*i*_ is the kinetic energy of the raindrop (J), *m*_*i*_ is the quality of the raindrop (J), *v*_*i*_ is the velocity of the raindrop (m/s), *d*_*i*_ is the raindrop diameter (mm), and *ρ* is the density of water (g/cm^3^).

Based on the total kinetic energy of the filter paper (e), the raindrop kinetic energy for every millimeter of rainfall per unit area can be calculated using the following formula (Eq 5):

$$E=(\frac{e}{S})/(\frac{M}{\rho S})=\frac{e\rho }{M}=\frac{e\rho }{\overset{i}{\underset{l}{\sum }}m_{i}}=\frac{e\rho }{\overset{i}{\underset{l}{\sum }}\frac{1}{6}\pi d_{i}^{3}\rho }=\frac{e}{\overset{i}{\underset{l}{\sum }}\frac{1}{6}\pi d_{i}^{3}}$$ (5)

where E is the raindrop kinetic energy of every millimeter of rainfall per unit area (J/m^2^/mm), and S is the area of the filter paper (m^2^). The value of E was 16.37 J/m^2^/mm under a rainfall intensity of 75 mm/h. The mid-value of the raindrop diameters (d_50_) was 1.5 mm, and 91.55% of the diameters were less than 1 mm. These results indicated that the simulated rainfall system could ensure that the kinetic energy of the simulated rainfall was maintained close to that of natural rainfall.

### Experimental materials and monitoring methods

The experiment materials included river sand and Lou soil. The river sand samples were collected from the middle and lower reaches of the Wei River bank in the Yangling District, and the Lou soil was also collected from Yangling District, Shaanxi Province, China [56]. The collected soil was air-dried and sieved through a series of corresponding opening sieves. The sediment bed for the experiment was composed of three layers, from bottom to top, of medium sand, fine sand and mixed soil. The layer thicknesses were 98.5, 1 and 0.5 cm, respectively. The bottom layer consisted of medium sand, and its bulk density was 1.4~1.5 g/cm^3^. The middle layer consisted of fine sand with a particle size of less than 0.25 mm, and the average soil bulk density was approximately 1.6 g/cm^3^. The top layer consisted of a mixture of Lou soil and fine sand, with a weight ratio of approximately 2:5 and a soil bulk density that was also approximately 1.6 g/cm^3^. The nitrogen fertilizer treatments in this study included two rates of fertilizer input: high (225 kg/ha NH_4_NO_3_) and control (no fertilizer input). The high rate was selected based on the internationally recognized safe limit of chemical fertilizer application (225 kg/ha). Ten minutes prior to rainfall onset in each fertilizer experiment, 112.5 g NH_4_NO_3_ was mixed with approximately 750 g surface soil and uniformly applied to the surface soil.

The primary parameters used to monitor water quantity were the amount of surface runoff, the time series surface flow, the groundwater flow, the groundwater level and the soil moisture, as described in detail in the literature [56]. The primary parameters used to monitor water quality were the nitrate concentrations in the surface flow and groundwater flow. Samples (50 ml) of the surface and ground water were collected at 10-min intervals during the experiments. A portable spectrophotometer (DR 2800, Hach, Loveland, Colorado) was used to measure the nitrate concentrations via the cadmium reduction method [57]. After the rainfall experiments, separate soil samples were collected using an earth drill at points of 0, 1, 2, 3, 4 and 5 m across the entire profile, and the measuring depths were 0–5, 5–20, 20–40, 40–60, 60–80 and 80–100 cm, respectively. The samples were analyzed by flow injection analysis (FIA STAR 5000) to determine the nitrate residues in the experimental soil.

### Ethics Statement

**Fig 4:**
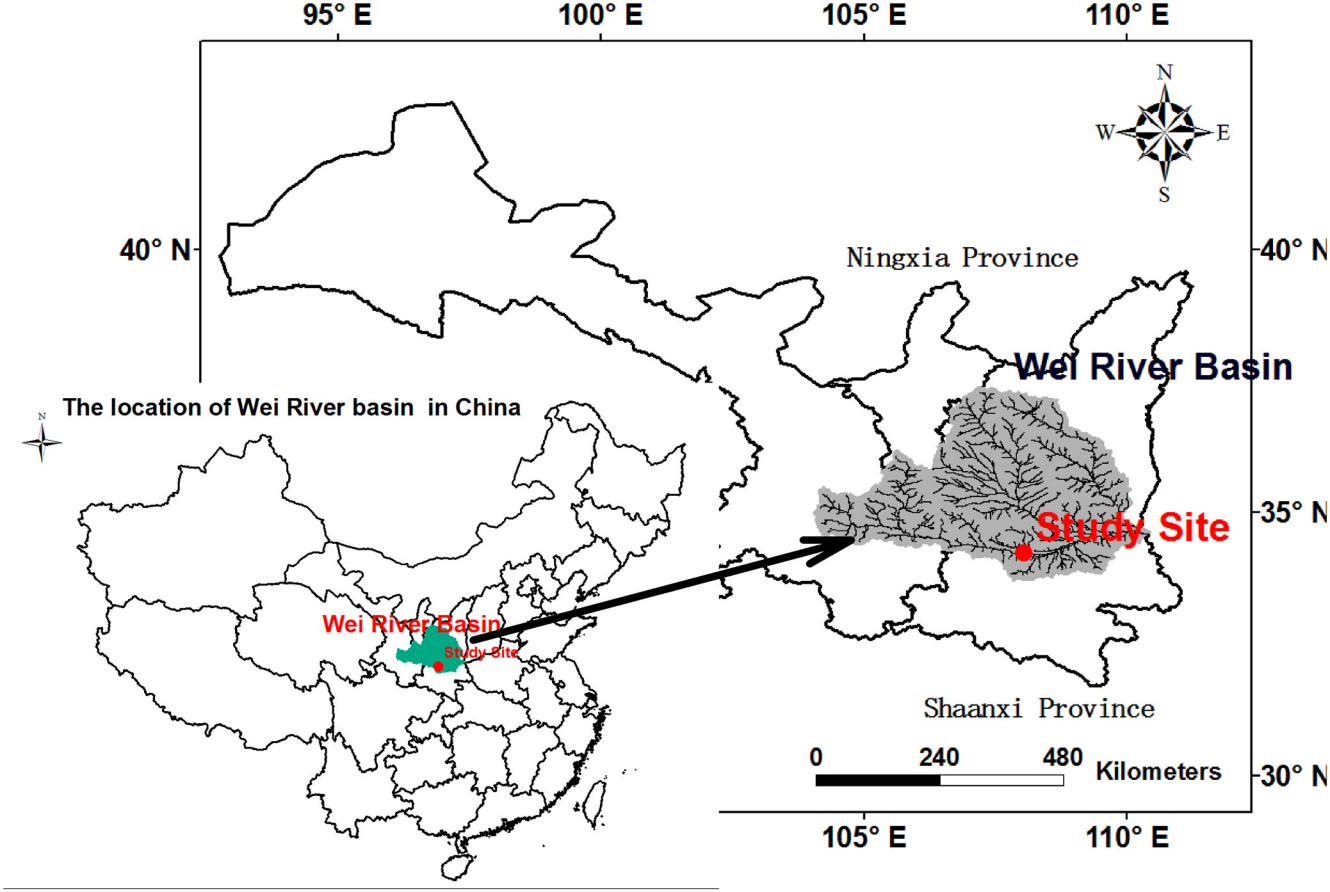
Location of the study site.

No specific permissions were required for these sampling activities because the location (as shown in Fig 4) is not privately owned or protected, and because the field activities did not involve endangered or protected species.

## Results and Discussion

### Time-series trend of surface and groundwater flow

During the experiments, most of the rainfall flowed out of the system as surface water, and the remainder of the rainfall infiltrated into the soil. The infiltrate was drained away primarily in the form of groundwater, and the remainder of the infiltrate was intercepted by the sediment bed. The amounts of cumulative surface and ground water for all twelve experiments were 869.75 mm and 450.44 mm, respectively. The results of the water balance of all twelve experiments showed that approximately 55.2% of the rainfall was lost through surface flow, approximately 28.6% recharged into groundwater, and the sediment bed retained approximately 16.2% of the water at the end of the last experiment.

**Fig 5:**
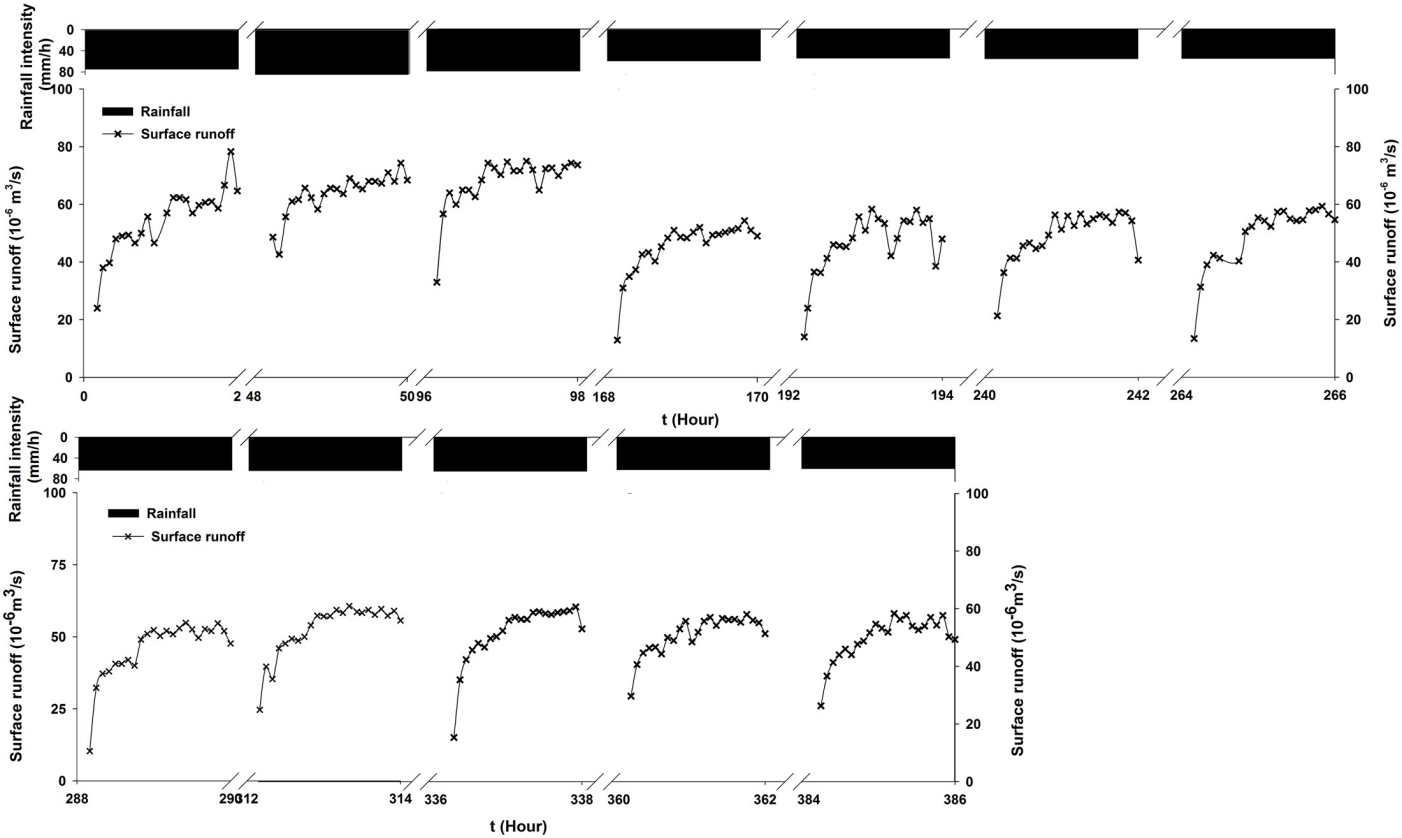
Time-series process of surface flow.

**Fig 6:**
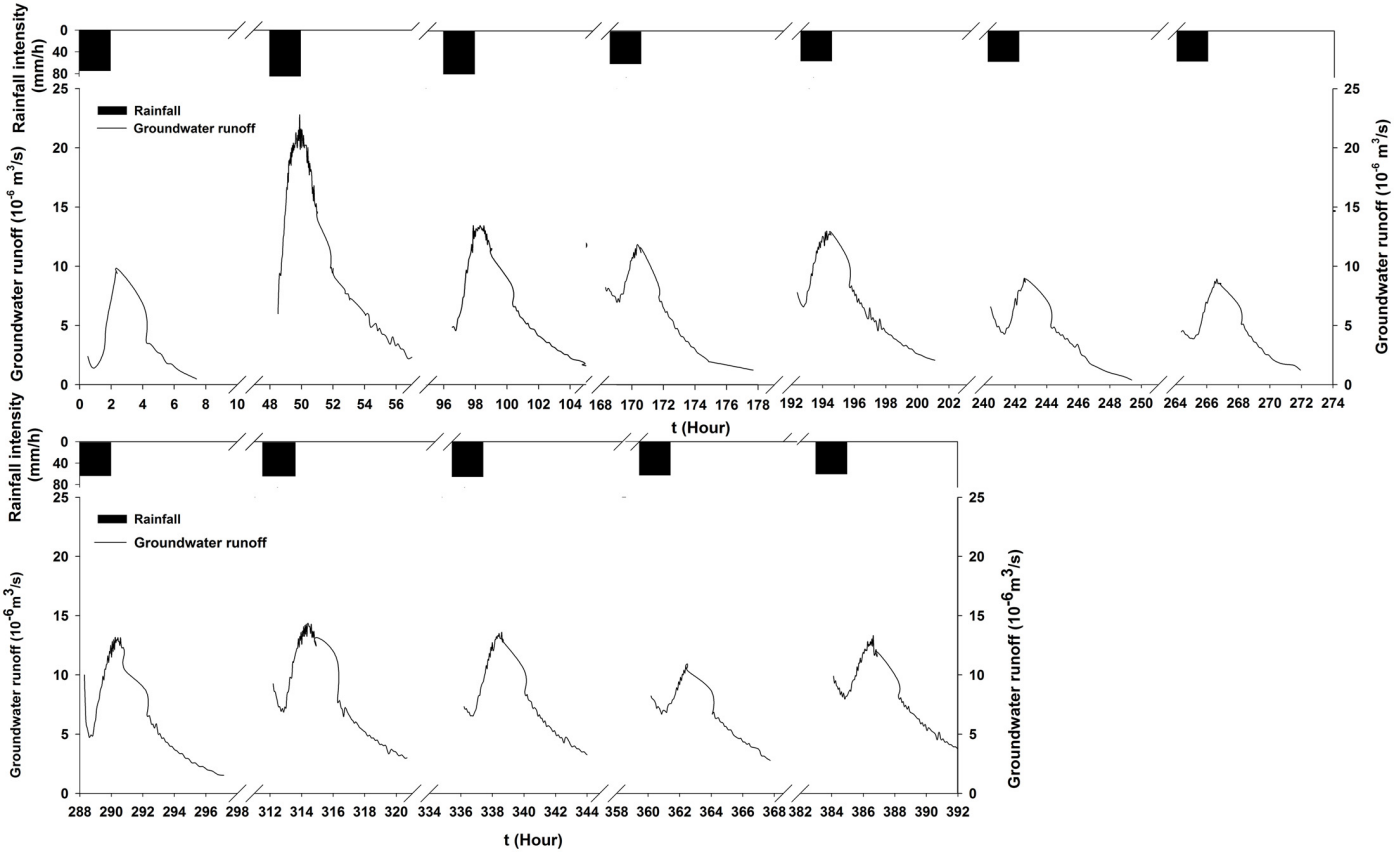
Time-series process of groundwater flow.

Fig 5 shows the time-series process of surface flow for all twelve experiments. As shown in this figure, all of the processes exhibited similar trends: they quickly increased in the first 10 minutes and then gradually stabilized, particularly after 50 minutes of rainfall. However, the ranges of increase were different due to the differences in the rainfall intensities. There were also significant differences in the magnitude of groundwater flow among the 12 experiments; however, all of the time-series processes of groundwater flow exhibited similar trends: they initially increased sharply with the rainfall duration but then gradually decreased after the rainfall terminates (Fig 6, S1 Table).

### Nitrate accumulation and leaching in surface and ground water

#### Nitrate loss with surface flow

**Fig 7:**
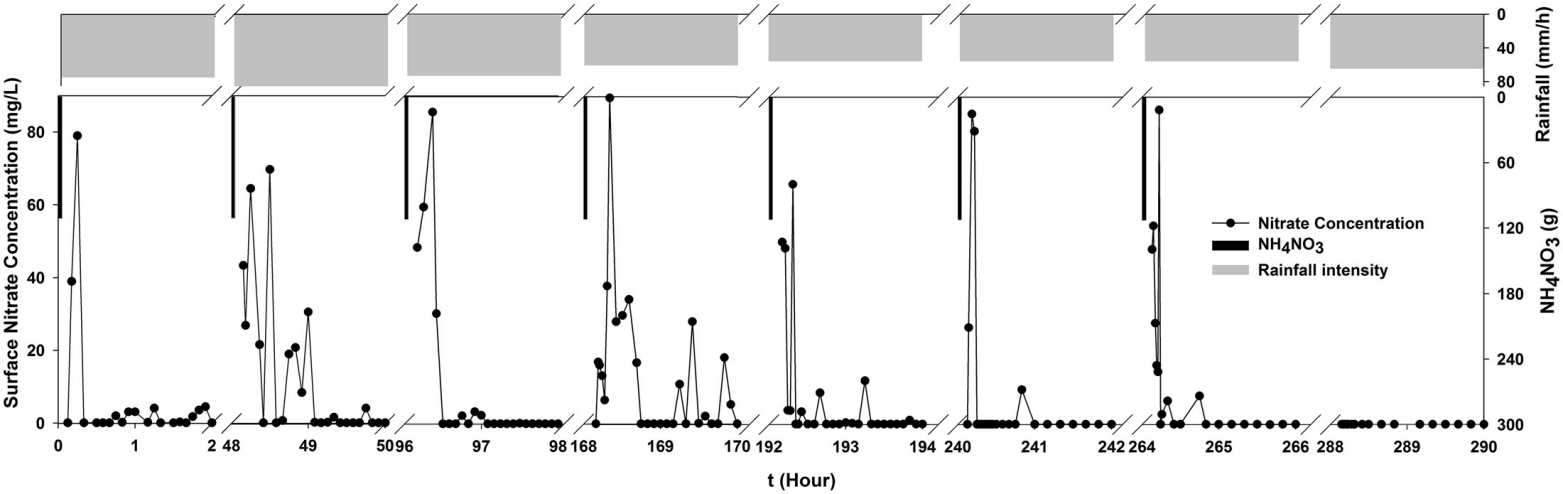
Time-series nitrate concentration in surface water.

Fig 7 shows the changes in nitrate concentration in surface water during the experiment (S2 Table). As shown in Fig 7, the the nitrate concentration increased quickly and then it rapidly decreased and gradually stabilized at a low value during every fertilization experiment. The nitrate fertilizer lost in with the surface water and the concentration peaked at about 19 minutes of rainfall, except for the 2nd and 4th experiments. The highest nitrate concentration was 89.7 mg/L, and the average maximum value for all 7 fertilization experiments was 80.3 mg/L. NO_3_^-^–N was mainly present in the infiltrated water. Its loss depended mainly on the amount of runoff, the rainfall intensity and the amount of interaction time between the surface runoff and soil particles [58]. This was in agreement with the nitrate fertilizer loss with groundwater and different from its loss with surface water in our study. Possibly because the nitrate fertilizer loss in surface water mainly occurred during the earlier stage of rainfall and there were no statistical significances among rainfall intensities during our experiments. Under simulated rainfall conditions, the water-soluble nitrogen loss with surface water flow has been reported to account for approximately 50% ~ 60% of the total nitrogen loss in the case of heavy rain after nitrogen application [59]. Under our simulated rainfall conditions, about 50.53% of the nitrate-nitrogen of the total fertilizer application was ramined in the experimental soil. Farmland nitrogen was transported into surface water by surface flow, which caused a substantial loss of soil nitrogen and was then drained away [35]. Contaminated water flowed into the field and entered into the river, which gradually transported nonpoint source pollution to the water body. Rainfall runoff is a mainly driving force of soil nitrogen loss. When no fertilizer was applied to the underlying surface, the nitrate was nearly undetectable in the surface runoff. As shown in Fig 7, the nitrate concentrations in the 8th experiment without fertilization were close to zero. The reduction of surface runoff and the available nitrogen in topsoil is a key to decreasing the loss of farmland nitrogen fertilizer. The nitrate concentrations quickly decreased to low values or even to zero during the rainfall process. Therefore, the earlier stage of rainfall was a crucial period for preventing nonpoint source pollution. Nonpoint sources follow a wide range of routes to aquatic environments and depend on the hydrological balance of overland flow, through flow and base flow [60].

#### Time-series trend of nitrate concentration in groundwater

**Fig 8:**
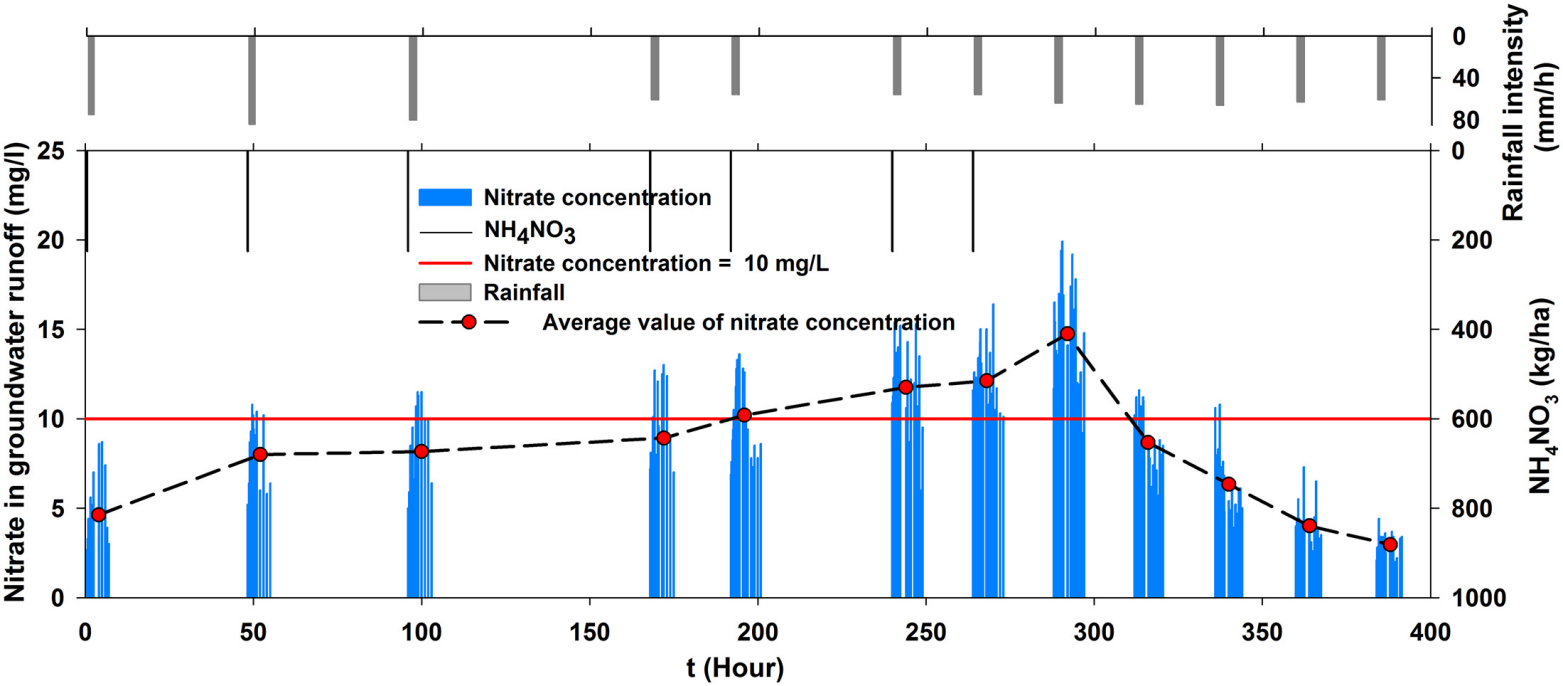
Time-series nitrate concentration in groundwater.

Under rainfall conditions, high levels of soluble nitrates (NO_3_^-^–N) leak through soil and into groundwater with infiltration flow and then drain off with groundwater flow [35]. Our rainfall experiments also showed that only a small part of nitrates lost with surface water and high levels of soluble nitrates leak with infiltration flow. As shown in Fig 8, the average nitrate concentrations of groundwater increased continuously from 1st to the 8th experiments (0~300 hours), and then they began to decrease from the 9th experiment (S3 Table). None of the values during the 1st experiment exceeded the limit of nitrate concentration potability (10 mg/L) [8–10]. The nitrate concentrations increased as the experiments progressed. The mean values and most of the measured values remained below 10 mg/L prior to the 5th experiment. In contrast,as the experiments progressed, the mean concentrations were greater than 10 mg/L, and this value increased with time. Prior to the 7th experiment, all of the concentrations were greater than 10 mg/L. Next, experiments without fertilization were performed starting from the 8th experiment. However, the concentrations in the 8th experiment increased continually, and this experiment presented the maximum concentration of all of the experiments, with an average value of 14.75 mg/L. This result indicated that a reduction in fertilizer application would not lead to a rapid decrease of nitrate concentrations in groundwater. Because nitrate accumulation is the premise of leaching, a substantial amount of nitrate must have accumulated in the soil in the prior experiments. Then, the infiltration flow provides a carrier for the accumulated NO_3_^-^–N in the soil profiles to move down, finally presenting the possibility of contaminating the groundwater [27, 61].

The nitrate concentrations thenbegan to decrease with the progression of the experiments with no fertilization. The rate of decrease in the mean concentration remained relatively constant at approximately 2 mg/L for every experiment. The average concentrations varied between 4.6 and 12.2 mg/L during the fertilization experiments, whereas they ranged from 2.9 to 8.6 mg/L for the experiments with no fertilization. Two conditions must be met for nitrate leaching. The first condition is nitrate accumulation in soil and the second condition is infiltration flow [35, 61]. Nitrate accumulation in soil increases as the nitrogen rate increases and is the premise of leaching. Rainfall and runoff are two primary driving forces of soil nitrogen loss.

#### Cumulative effects of nitrate in groundwater

**Fig 9:**
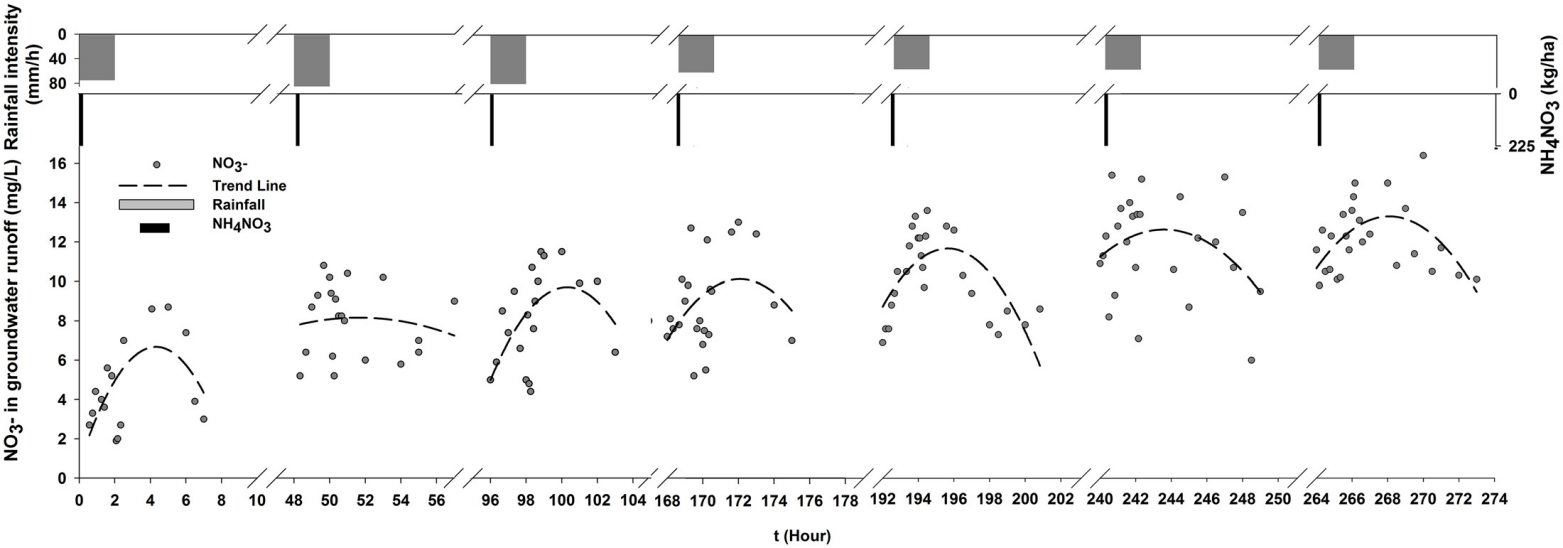
Time-series nitrate concentration in groundwater with fertilization.

Fig 9 shows the time-series nitrate concentration of groundwater under fertilization, rainfall and groundwater runoff conditions. The nitrate concentration showed a general increasing trend throughout the process. The average concentration ranged from 4.63 mg/L—12.13 mg/L, which represented a 2.62-fold increase. As shown in Fig 9, there was a positive correlation between rainfall intensity and groundwater flow. When the rainfall intensity was larger and the runoff was higher, the added nitrate concentration was greater. From the 1st experiment to the 2nd experiment, the concentration increased by 3.36 mg/L, and the rate of increase remained relatively constant at approximately 1.13 mg/L for the other experiments. The second experiment had the largest rainfall intensity, which offered the strongest driving force for soil nitrogen loss. These results demonstrated that deep percolation and nitrate leaching most likely occurred following a heavy precipitation event [62]. Furthermore, high precipitation increasedboth the amount of nitrate N in runoff and could increase the negative impact on water quality [63].

In contrast to the overall trend of nitrate concentration, the time series of groundwater flow increased until the rainfall stopped, followed by a gradual decrease during one experiment. The nitrate concentrations also exhibited the same parabolic trend. Initially, the nitrate concentration increased as the groundwater flow increased because the large infiltration flow provided support for the highly mobile nitrate [7, 64, 65]. Then, the nitrate concentration decreased because the infiltration flow for NO_3_^-^ transportation had been decreasing for a while [66]. The delay time in which the groundwater runoff and nitrate concentrations decreased was approximately 0.94 hours. The change in the nitrate concentration lagged behind the flow for nitrate accumulation. The difference between the highest and the lowest concentrations recorded in one experiment greater than 5.2 mg/L.

**Fig 10:**
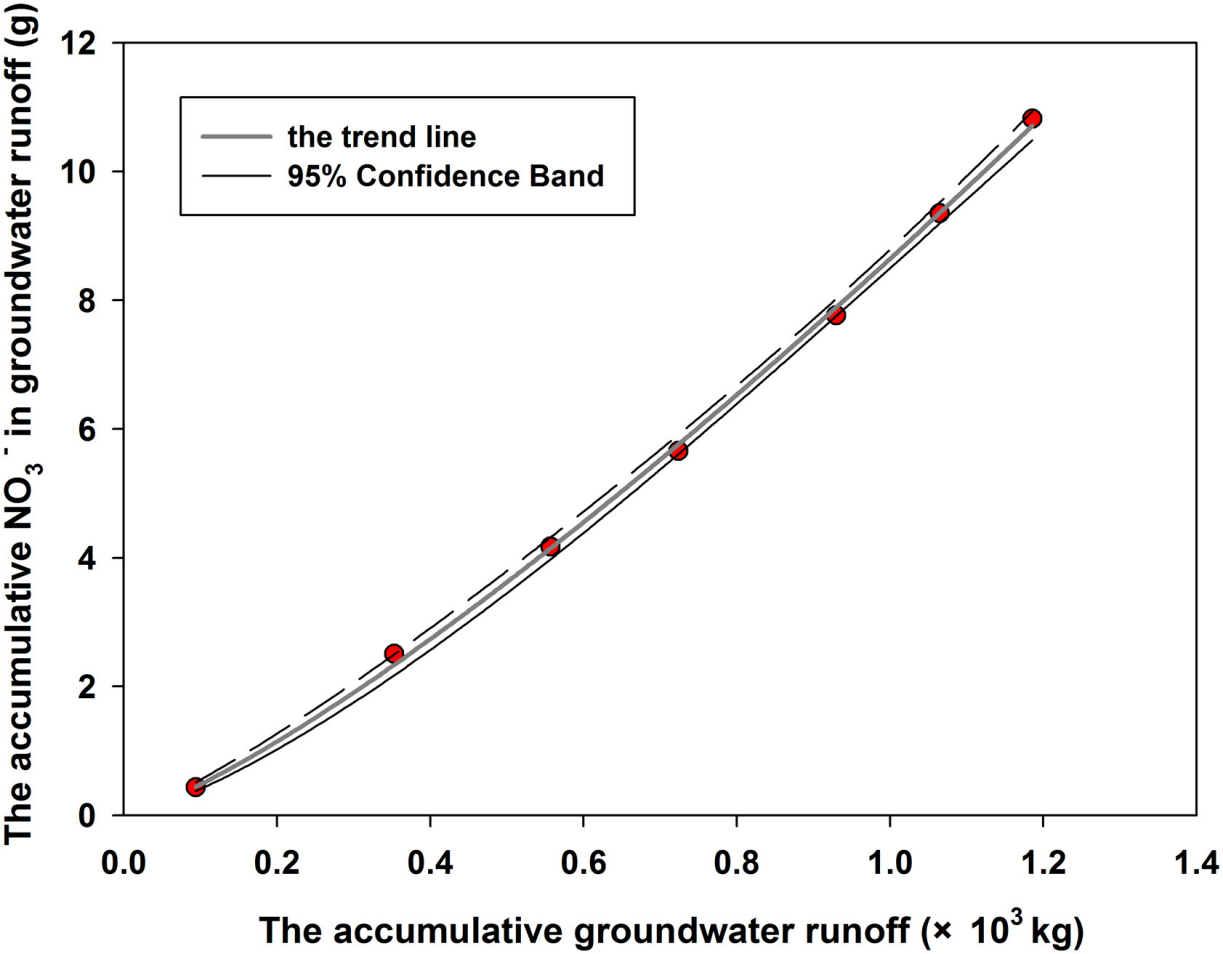
Relationship between nitrate concentrations and groundwater runoff.

A positive relationship between the accumulative groundwater runoff and the accumulative nitrate content was obtained as follows (Fig 10, i S4 Table):

$$M=8.68m^{1.26}$$ (6)

where M and m denote the loss amount of nitrate in groundwater (g) and groundwater runoff (kg), respectively. The nitrate content was low for small runoff and increased relatively rapidly for high runoff. M and m were described by a positive power function, with a correlation coefficient of 0.9996. These findings further confirmed that groundwater recharge aided nitrate leaching. Many studies have also shown that the nitrate concentration in groundwater increased as the groundwater recharge increased in shallow aquifers [12, 15, 42, 67]. It could be concluded that groundwater recharge should be controlled in areas of high rainfall to minimize nitrate leaching, thus reducing the risk of groundwater contamination.

#### Leaching effects of nitrate in groundwater

**Fig 11:**
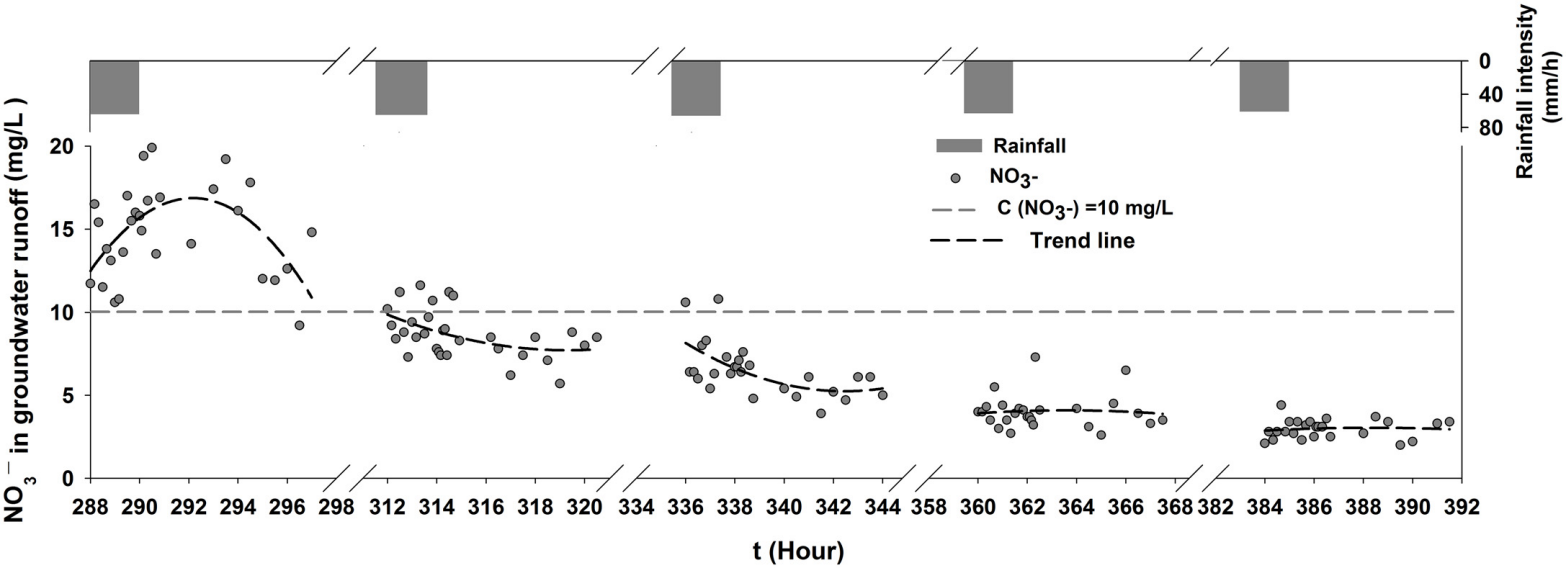
Time-series nitrate concentration and flow of groundwater with no fertilization.

Fig 11 shows the time-series nitrate concentrations of groundwater with no fertilization under rainfall and groundwater runoff conditions. The nitrate concentration exhibited a general decreasing trend throughout the experimental process. The average concentration range was 14.75 mg/L—2.97 mg/L, which represented a 4.97-fold decrease. The concentrations steadily decreased at a mean rate of 2 mg/L in every experiment. The time-series concentration of the 8th experiment in which no fertilizer was applied, had the same parabolic trend as the results of the fertilization experiments. These results indicated that large amounts of accumulated NO_3_^-^–N in the soil profiles continuously moved down with the flow, ultimately causing more serious groundwater contamination. Then, the groundwater flow remained high, but the nitrate concentration changed to a decreasing trend in the subsequent experiments with no fertilization because the soil nitrate was virtually moved after several successive rainfalls and was limited in its ability to produce a high rate of nitrate leaching to groundwater. The concentration of groundwater nitrate stabilized at low values throughout the entire process. Therefore, without considering microorganisms and nitrification / denitrification processes, nitrate leaching requires two primary conditions: a significant concentration of nitrate in soil water and sufficient rainfall or irrigation [17, 66]. When soil moisture is not a limiting factor, the excessive application of nitrogen fertilizer might lead to significant nitrate leaching [61, 68–70].

### Nitrate residues in experimental soil

**Fig 12:**
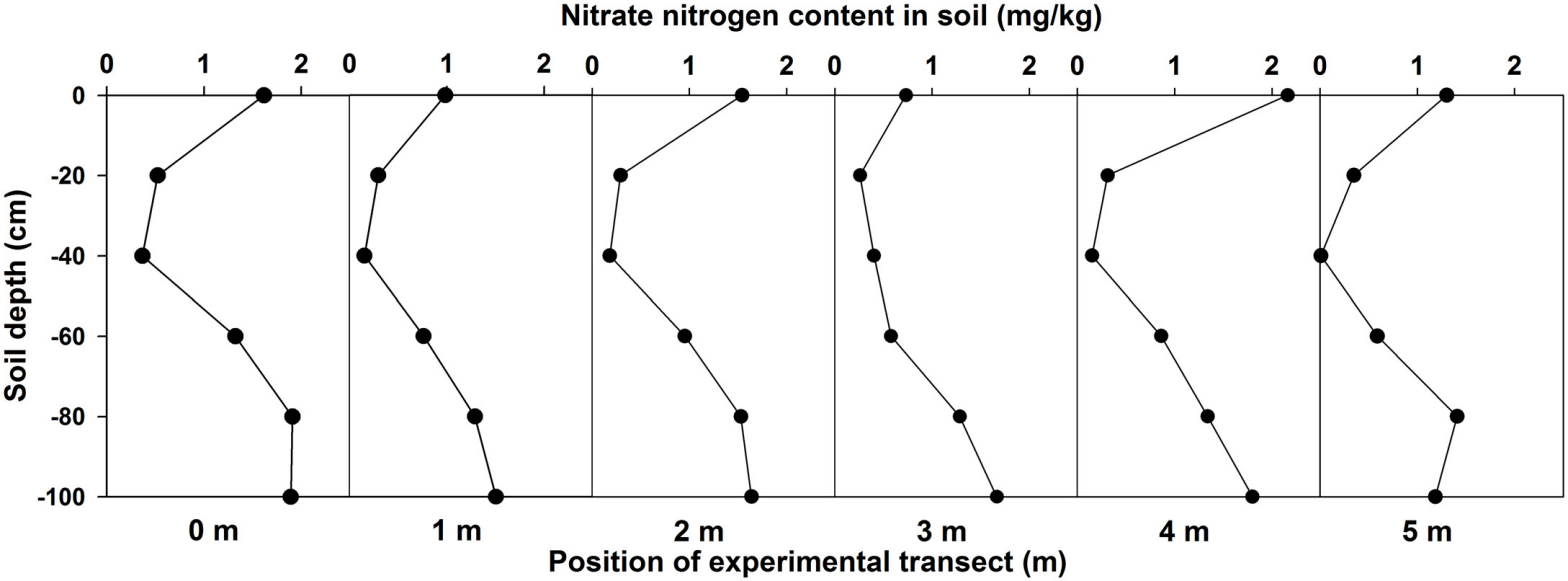
Nitrate residues in each layer of experimental soil.

Eight days after the rainfall experiments, the nitrate residues in the experimental soil were separately determined at points of 0, 1, 2, 3, 4, and 5 m across the entire profile, and the measuring depths were 0–5, 5–20, 20–40, 40–60, 60–80 and 80–100 cm, respectively. Based on this determination, the residues of nitrate-nitrogen content in the experimental soil were calculated. The result was 69.64 g, accounting for 50.53% of the nitrate-nitrogen of the total fertilizer application. Fig 12 shows the measurement results of nitrate-nitrogen in each soil layer. As shown in this figure, the content of nitrate = nitrogen initially increased and then decreased from the soil surface downward. The derived percentage distribution of the NO_3_-N residue in the soil for each layer can be obtained based on the results of the stratified statistics of the nitrate-nitrogen content, as shown in Table 2. The minimum residual of the soil nitrogen content was at a depth of 20–40 cm, accounting for only 4.65%, and the highest residual was at a depth of 80~100 cm, accounting for 35.12%.

**Table 2:** Distribution of NO_3_-N residues in the soil for the non-uniform soil layer.

Depth (cm)	Nitrate nitrogen content %
0–5	7.41
5–20	5.16
20–40	4.65
40–60	17.70
60–80	30.61
80–100	35.12

## Conclusions

Globally, nitrate is one of the most common groundwater contaminants and is primarily introduced into the environment from agricultural activities related to excessive use of nitrate-containing fertilizers and manure [71]. The effects of fertilizer application on the accumulation and leaching of nitrate with water flow were studied through simulated rainfall experiments under the following conditions: land use was a 3° bare slope and the soil layers consisted of medium sand, fine sand and mixed soil from bottom to top. For the water quantity, approximately 55.2% of the rainfall was lost through surface runoff, and approximately 28.6% was recharged into groundwater during one experiment. The surface flow quickly increased in the first 10 minutes and then gradually stabilized, particularly after 50 minutes of rainfall. The groundwater flow initially increased sharply but then gradually decreased after rainfall termination.

Regarding water quality, the nitrate concentration in the surface flow initially increased quickly, and then it rapidly decreased rapidly and stabilized at a low value. The nitrogen loss primarily occurred during the first 18.6 minutes of rainfall, and the rainfall runoff was the main driving force for soil nitrogen loss. The nitrate concentrations in the groundwater accumulated, and they were greater than 10 mg/L throughout the process until the 7th experiment. The nitrate concentration in the 8th experiment continually increased, although no fertilizer was applied from the 8th to 12th experiments. The nitrate concentration decreased in the subsequent experiments.

In terms of soil quality, 8 days after the 12 rainfall experiments, the nitrate residues in the experimental soil accounted for 50.53% of the nitrate-nitrogen of the total fertilizer application. The minimum residual of the soil nitrogen content was at a depth of 20–40 cm of soil layer.

It could be concluded that the earlier stage of rainfall is a crucial period for controlling the loss of farmland nitrogen fertilizer. The nitrate in groundwater accumulated quickly, and most of it remained in the soil, even after leaching by copious amounts of rain. Most of the nitrate residues existed at the surface and the bottom layers of the soil, presenting potentially more dangerous pollution for surface and ground water.

## Supporting Information

S1 Table Time-series groundwater runoff. (XLSX)

S2 Table Time-series nitrate in surface runoff. (XLSX)

S3 Table Time-series nitrate in groundwater runoff. (XLSX)

S4 Table Accumulative runoff vs-accumulative NO_3_- in groundwater. (XLSX)
